# Network pharmacology identify intersection genes of quercetin and Alzheimer’s disease as potential therapeutic targets

**DOI:** 10.3389/fnagi.2022.902092

**Published:** 2022-08-23

**Authors:** Caihui Wei, Shu Li, Yu Zhu, Wenzhi Chen, Cheng Li, Renshi Xu

**Affiliations:** ^1^Department of Neurology, Jiangxi Provincial People’s Hospital, Medical College of Nanchang University, Nanchang, China; ^2^Department of Neurology, The First Affiliated Hospital of Nanchang Medical College, Jiangxi Provincial People’s Hospital, Nanchang, China

**Keywords:** Alzheimer’s disease, quercetin, DYRK1A, NOS2, immune infiltration

## Abstract

**Background:**

Currently, there are no efficient therapies for Alzheimer’s disease (AD) among the elderly, although it is the most common etiology of dementia among the elderly. Quercetin, which has a variety of therapeutic properties, may pave the way for novel approaches to AD treatment. In the AD patients’ frontal cortex, current study aims to identify the potential mechanisms of quercetin’s pharmacological targets.

**Materials and methods:**

The pharmacological targets of quercetin have been studied from DrugBank and SwissTarget. In order to distinguish AD-associated genes targeted by quercetin (Q-ADGs), we utilized an integrated intersection of gene expressions of the frontal cortex in combination with transcriptome analysis. To detect cortex-related Q-ADGs and immune-related Q-ADGs, a drug screening database and the immune infiltration analysis was utilized. The Q-ADGs were then linked with the AD severity scores (MMSE scores) to find severity-associated Q-ADGs. In addition, the miRNA-seq datasets were examined to identify severity-associated Q-ADG-miRNAs. Twelve genes, more frequently related to AD by previous studies among all the genes identified in the present study, were subjected to the verification of qRT-PCR in AD cell model.

**Results:**

In the frontal lobe of AD, 207 Q-ADGs were discovered and found that axonogenesis, glial differentiation, and other biological processes had been enriched. There were 155 immune-related Q-ADGs (e.g., COX2, NOS2, HMGB1) and 65 cortex-related Q-ADGs (e.g., FOXO1, CXCL16, NOTCH3). Sixteen Q-ADGs (e.g., STAT3, RORA, BCL6) and 28 miRNAs (e.g., miR-142-5p, miR-17-5p) were found to be related to MMSE scores. In the qRT-PCR results, six out of twelve genes were significantly regulated by quercetin. DYRK1A, FOXO1, NOS2, NGF, NQO1, and RORA genes were novel target of quercetin in AD. DYRK1A, NOS2, and NQO1 genes targeted by quercetin have benefits in the treatment of AD. However, FOXO1, NGF, and RORA genes targeted by quercetin might have a negative impact on AD.

**Conclusion:**

The role of quercetin in AD appears to be multifaceted, and it can affect patients’ frontal cortex in a variety of pathways, such as axonogenesis, immune infiltration, and glial cell differentiation. DYRK1A, NOS2, and NQO1 might be potential novel effective drug targets for quercetin in AD.

## Introduction

People with Alzheimer’s disease (AD) suffer gradually declining cognitive function, personality changes, and psychiatric symptoms. AD is the leading cause of age-related dementia worldwide (Wang et al., 2009). There is still uncertainty as to how AD develops, since it is a complicated neurodegenerative disorder (Wang et al., 2018). However, genetic changes may be prevalent causes of early-onset familial AD (FAD), such as mutation in the presenilin or amyloid precursor protein (APP) gene causing the most frequent and aggressive forms of FAD (Athan et al., 2001; Dewachter and Van Leuven, 2002; Bergmans and De Strooper, 2010). At the genetic level, significant progress has been made in understanding AD’s etiology (Damjanac et al., 2009; Campion et al., 2019; Ułamek-Kozioł et al., 2020; Zhang X. et al., 2020). To date, AD is mostly resistant to conventional therapies. Therefore, therapeutic drug may be developed targeting AD on the basis of pathological, biochemical and genetic evidence.

Quercetin, a plant-derived flavonoid, has been found as a promising antioxidant and antiinflammatory molecule (Katsarou et al., 2000; Panicker et al., 2010). Quercetin has been demonstrated to decrease the immune response in a variety of immunological-related disorders, including experimental allergic encephalomyelitis (EAE) and even infection with COVID-19 (Muthian and Bright, 2004; Zheng et al., 2021). Quercetin’s efficacy in treating experimental EAE is often linked to its ability to inhibit Th1 differentiation and IL-12 signaling (Muthian and Bright, 2004). Additionally, quercetin has therapeutic benefits on neurodegenerative illnesses by modulating a variety of proteins including Bcl-2, PARP, Bax, COX2, NF-kB, STAT3, chemokines, and cytokines (Ghafouri-Fard et al., 2021; Zingales et al., 2021). Recently, quercetin has been shown to prevent pathologies and promote neuroprotective effect in the therapy of AD by relieving functional and cognitive symptoms (Qi et al., 2020). *In vivo* and *in vitro*, numerous investigations shown that quercetin improves the clearance of aberrant proteins, such as β-amyloid and hyperphosphorylated tau, which are important pathologic markers of AD (Suganthy et al., 2016; Lu et al., 2018; Qi et al., 2020). A lipophilic compound known as quercetin penetrated the blood-brain barrier (BBB) in experiments with animal models of degenerative diseases (M S and C D, 2017; Orhan, 2021). This indicates quercetin has direct effects on the central nervous system (CNS). A number of studies have implicated quercetin in a number of activities, including mitophagy, inhibition of microglia and astrocyte activation, regulation of the inflammatory response in dendritic cells, and inhibition of neuronal death (Han et al., 2021). Given that these processes of pathophysiology had been identified in AD patients, it was hypothesized that quercetin may also have therapeutic benefits on AD patients’ pathogenic processes.

Recently, more and more novel approaches, such as Connectivity Map and microarray data (Iyaswamy et al., 2020), have been used to identify drug therapeutic targets targeting AD. Until yet, most of previous research on AD animal models studied on how quercetin had exerted its impact on the metabolism of the aggregation of β-amyloid protein and APP (Halevas et al., 2020; Yu et al., 2020). However, a lot of AD models do not accurately mimic real illness, especially late-onset disease. As a result, direct study on human brain tissue is required for AD research. However, there is presently no evidence proving quercetin’s function in AD patients’ brain tissue. The network pharmacology provides a novel tool for identifying successful medication components and pathways, as well as a more complete comprehension of the pharmacological effects of pharmaceuticals in particular disorders. Additionally, this approach enables direct examination of brain tissue. The network pharmacological approach was utilized in this research to investigate the possible pharmacological targets and mechanism of quercetin in the treatment of AD.

## Materials and methods

### Study design

**FIGURE 1:**
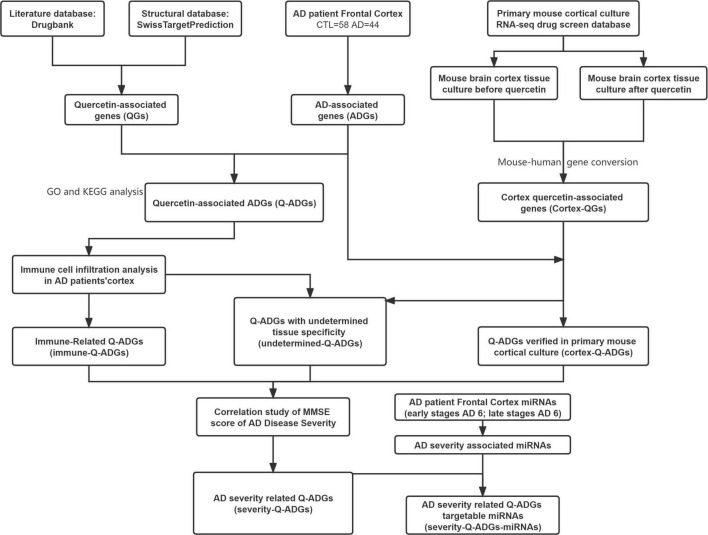
Flowchart of the effect of quercetin on AD. AD, Alzheimer’s disease; CTL, healthy control; ADGs, AD-associated genes; QGs, quercetin-associated genes; Q-ADGs, quercetin-associated AD-associated genes.

DrugBank and SwissTarget were used to obtain the pharmacological macromolecular targets of quercetin (i.e., quercetin-associated genes, QGs). We performed an integrated intersection of the gene expressions in frontal cortex (44 AD patients and 59 healthy controls) to detect AD-associated genes (ADGs) and quercetin-associated genes for AD (Q-ADGs). To distinguish immune-related and cortex-related Q-ADGs, we used a drug screening database and immune cell infiltration analysis. To detect severity-related Q-ADGs, the levels of Q-ADG expression were associated with Mini-Mental State Exam (MMSE) scores of AD. Additionally, to distinguish severity-related Q-ADG-miRNAs, miRNAs was predicted to interact with severity-related Q-ADGs. The flow chart of network pharmacology analyses is shown in Figure 1. We employed the same method in this investigation to evaluate frontal cortex, entorhinal cortex, and temporal cortex data. The article’s primary material relied heavily on frontal cortical data. Supplementary Tables 2, 3 include entorhinal cortex and temporal cortex data. Twelve genes, more frequently related to AD by previous studies among all the genes identified in the present study, were subjected to the verification of qRT-PCR.

### Quercetin-associated genes

PubChem (Kim et al., 2019) was used to acquire the chemical structures of quercetin. Experimentally validated quercetin drug targets was utilized to obtained from the drug target database DrugBank (Wishart et al., 2018). SwissTargetPrediction (Daina et al., 2019) was used to screen quercetin targets based on its chemical structures, with a probability of 1.0 implying the retrieval of an experimentally validated bioactivity rather than a prediction. The name of pharmacological targets was standardized by UniProt (The UniProt Consortium, 2015). With STRING (Szklarczyk et al., 2021), with the confidence criterion set to high confidence and the species criterion set to Homo sapiens, a network of protein-protein interaction (PPI) was constructed.

### Alzheimer’s disease-associated genes

In this study, the microarray dataset for AD patients was accessed from the Gene Expression Omnibus database (Barrett et al., 2012), a worldwide repository for high-throughput gene expression datasets. The frontal cortex was analyzed in detail and without redundancy using two microarray datasets (GSE48350 and GSE5281) consisting of 44 AD patients and 59 healthy counterparts. The two datasets were both from Affymetrix Human Genome U133 Plus 2.0 Array which was subjected to the annotation for the two datasets. Then, the two datasets were processed by log2 transformation, merged under no outlier values to exclude abnormal genes, subjected to batch removal by using the ComBat function in R package sva (version 3.40.0), and quantile normalized on the linear scale using the normalize.quantiles function in R package preprocessCore (version 1.54.0). To identify ADGs, differentially expressed gene (DEG) analysis was conducted between AD patients and healthy controls. In order to correct multiple testing, the Benjamini-Hochberg method was applied in order to carry out the DEG analysis in the R package limma. Significant ADGs were distinguished with the conditions of | fold change | > 1.3 and p.adj < 0.05.

### Quercetin-associated Alzheimer’s disease-associated genes

The QGs and ADGs were intersected to determine quercetin-associated ADGs (Q-ADGs). The R package of clusterProfiler (version 4.0.5) (Yu et al., 2012) was used to examine these Q-ADGs for Gene Ontology (GO) analysis and Kyoto Encyclopedia of Genes and Genomes (KEGG) analysis. The enrichment analysis employed the following parameters: p.adj method: BH; p.adj 0.05; and significance level: Top 20. The PPI study was performed using STRING (version 11.5). After removing duplicates, confidence scores greater than 0.7 was used to create the PPI network. Cytoscape’s Cytohubba plug-in was employed to visualize hub genes from the PPI network.

### Immune infiltration in the frontal cortex

The expression matrix of immune cell subtypes in each AD patient’s brain could be deconvolved using CIBERSORT (Newman et al., 2019). One hundred and two samples were selected to perform a 1,000-permutation process using the default signature matrix in CIBERSORT to convert the gene expressions to 22 immune cell fractions data (*P* < 0.05). The correlation analysis of Pearson’s coefficient was employed to explore the relation between the Q-ADG expression and immune cell infiltrations in AD (*P* < 0.05).

### Cortex-related quercetin-associated genes and cortex-related quercetin-associated Alzheimer’s disease-associated genes

For a better understanding of quercetin’s direct effects on the cerebral cortex as well as the removal of disturbances caused by immune infiltration within the brain, we utilized the drug datasets (Hadwen et al., 2018) which contains transcriptomic data from multidrug-treated cerebrocortical cultures. As a result of comparison of the gene expression profile before and after quercetin administration using the drug screening database, we identified mouse cortex-related quercetin-related genes (cortex-related QGs) with the conditions of | Zscore | > 3 and *p* < 0.05 (Hadwen et al., 2018). Later, the R package biomaRt was utilized to convert the mouse gene sets to their human counterparts. To identify cortex-related Q-ADGs, the intersection of cortex-related QGs and ADGs was conducted.

### Undetermined quercetin-associated Alzheimer’s disease-associated genes

Undetermined Q-ADGs were those specific Q-ADGs that were not categorized as cortex-related Q-ADGs or immune-related Q-ADGs. The PPI network was created using the abovementioned criteria, and genes were analyzed using the GO functional classification system.

### Quercetin-associated Alzheimer’s disease-associated gene expression in clinical severity of Alzheimer’s disease

Ten AD patients were chosen with the criteria of MMSE scores ranging 11–26, which means the clinical severity of normal to moderate dementia. To identify severity-related Q-ADGs, the correlation analysis of Pearson’s coefficient was employed to investigate the relation between the MMSE scores and Q-ADG (cortex-related Q-ADGs, immune-related Q-ADGs and undetermined Q-ADGs) expressions (*P* < 0.05).

### Severity-related quercetin-associated Alzheimer’s disease-associated genes-miRNAs

The frontal cortex miRNA sequencing dataset was downloaded from GEO (GSE48552), consisting of 12 AD patient. DESeq2 object was initialized from raw reads count matrix by using readscount2deseq function in R package ImageGP (version 0.1.0). DEGs were acquired by using readscount2deseq as well. Normalized expressions were acquired by using deseq2normalizedExpr function in R package ImageGP with log2 transformation for further analysis. ImageGP was based on R package DESeq2. Significant ADGs were distinguished with the conditions of | fold change | > 1.5 and p.adj < 0.05. By comparing conserved 6-mer, 7-mer, and 8-mer regions within each miRNA seed regions, TargetScan (Lewis et al., 2005) is used to identify potential miRNAs targeted by severity-related Q-ADGs. In the following step, to identify the severity-related Q-ADG-miRNAs, we intersected severity-related miRNA with potential targeted miRNAs.

### Cell culturing and reagents

From the Cell Bank^1^ (Serial: TCR 9), a differentiated PC12 cell line (#RRID:CVCL_F659) was purchased. We cultured PC12 cells in high glucose Dulbecco’s modified Eagle medium (DMEM; VivaCell, Beit Hae-mek, Israel) containing 1% penicillin/streptomycin (Solarbio, Beijing, China) and 10% fetal bovine serum (ExCell Bio, Shanghai, China) at 37°C and 5% CO2. Amyloid beta-peptide (25–35) (Aβ25-35; 98% peptide purity) was purchased from Chinapeptides (Shanghai, China). Quercetin (98% purity by HPLC) was purchased from Solarbio. Aggregated Aβ_25–35_ (final concentration 25 μM) (Wang and Xu, 2019) was included in the medium with or without quercetin (final concentration 80 μM) in the PC12 cell for 24 h (Yu et al., 2020).

### Quantitative real-time polymerase chain reaction

An RNA isolation reagent (CAT: G3013) was used to isolate total RNA from PC12 cell line. Based on the manufacturer’s instructions, RNA was reverse transcribed into cDNA using a RT First Strand cDNA Synthesis Kit (CAT: G3330). Twelve genes, more frequently related to AD by previous studies among all the genes identified in the present study, were subjected to the verification of qRT-PCR (Supplementary Table 9). QRT-PCR was carried out using 2 × SYBR Green qPCR Master Mix (None ROX) (CAT: G3320) with primers. The 2^–ΔΔCt^ method was used to quantify mRNA relative expression levels with GAPDH as the internal control. All reagents were purchased from Servicebio (Wuhan, China). At least three independent experiments with triplicate samples are required for statistical analysis of qRT-PCR data.

### Statistical analysis

Correlation analyses were under the Pearson correlation. R > 0.7 denoted a very high linear correlation, 0.5 < R ≤ 0.7 a substantial linear correlation, 0.3 < R ≤ 0.5 a weak linear correlation, and R ≤ 0.3 denoted no linear connection. For non-normal distributions, the Mann-Whitney test was used instead of unpaired t-tests. Results were shown as mean ± SEM. mRNA relative levels were demonstrated on linear scale as 2^–ΔΔCT^ means ± SEM. P < 0.05 was considered statistically significant.

## Results

### Quercetin-associated genes

A total of 59 verified pharmacological targets for quercetin were found using the SwissTarget database and 32 using the DrugBank database. As shown by the PPI network, 2400 proteins interacting with the drug targets were discovered and named QGs (Supplementary Table 1).

### Alzheimer’s disease-associated genes

Compared to a healthy control group, we found 3,285 ADGs in the frontal cortex of AD patients. Among these genes, 1,624 of them were upregulated, and 1,661 of them were downregulated (Supplementary Table 2). Simultaneously, the same analysis was conducted on data from AD patients’ temporal and entorhinal cortexes (Supplementary Table 2 and Supplementary Figure 1).

### Quercetin-associated-Alzheimer’s disease-associated genes

**FIGURE 2:**
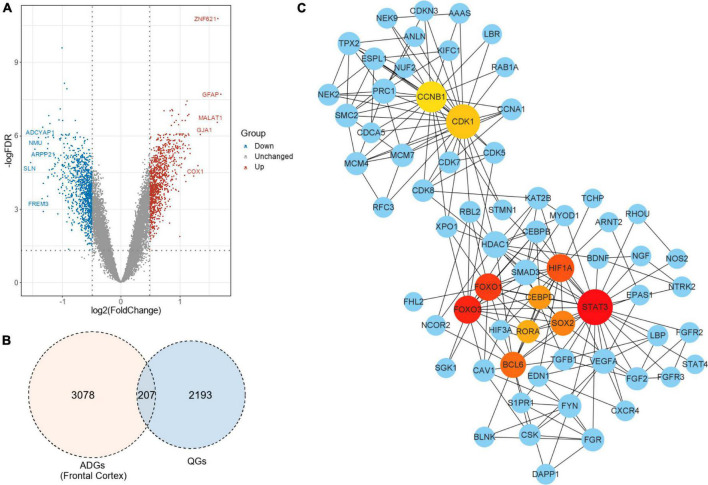
Drug targets of quercetin and quercetin-associated Alzheimer’s disease-associated genes (Q-ADGs). **(A)** As compared with healthy controls, the volcano plot illustrates the differentially expressed genes (DEGs) in the frontal cortex of AD patients. Red highlights indicate genes that have been upregulated significantly. Blue highlights indicate genes that have been downregulated significantly. Gray colors indicate genes that are unchanged. **(B)** QGs, ADGs, and Q-ADGs are demonstrated in Venn diagram. **(C)** Q-ADGs interact with each other in the PPI network whose each node represents a Q-ADG.

In the frontal cortex, 207 genes were QGs (116 upregulated and 91 downregulated) among the 2,287 ADGs detected. As a result, these QGs were quercetin target and were named Q-ADGs. In the PPI network, 619 interacting edges were formed, containing 207 Q-ADGs (Figure 2 and Supplementary Table 3).

### Gene ontology and kyoto encyclopedia of genes and genomes pathway of quercetin-associated-Alzheimer’s disease-associated genes

**FIGURE 3:**
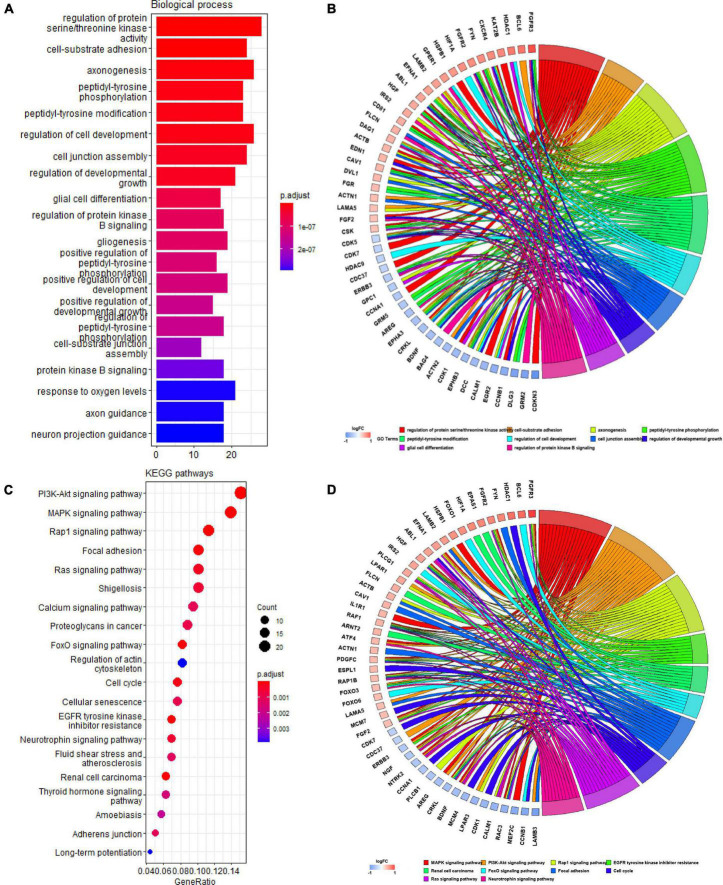
Gene ontology (GO) and kyoto encyclopedia of genes and genomes (KEGG) analysis of quercetin-associated Alzheimer’s disease-associated genes (Q-ADGs). **(A)** Bar plot shows GO enrichment of Q-ADGs. **(B)** Chord plot shows the genes are linked *via* ribbons to their assigned GO terms. **(C)** Dot plot shows KEGG pathways of Q-ADGs. **(D)** Chord plot shows the genes are linked *via* ribbons to their assigned KEGG pathways.

We identified 207 Q-ADGs in the frontal cortex, whose biological processes were enriched in, for example, axonogenesis, glial cell differentiation, gliogenesis, axon guidance, neuron projection guidance. Additionally, these Q-ADGs enriched in KEGG pathways such as MAPK signaling pathway, PI3K-Akt signaling pathway, axon guidance, and neurotrophin signaling pathway (Figure 3 and Supplementary Table 4).

### Immune-related quercetin-associated-Alzheimer’s disease-associated genes

**FIGURE 4:**
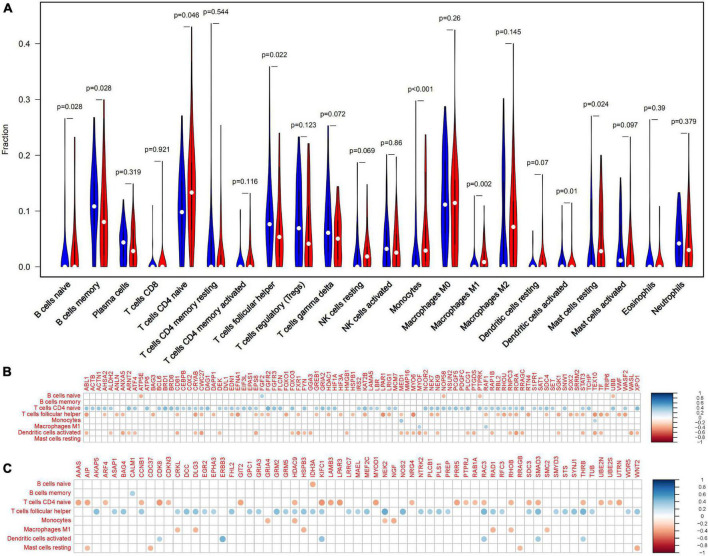
Immune-related quercetin-associated Alzheimer’s disease-associated genes (Q-ADGs). **(A)** The Violin plot illustrates the infiltration of immune cells in AD patients’ frontal cortex. **(B)** Analysis of the correlation between immune cells signals and the expressions of upregulated Q-ADGs. The correlation coefficient is represented by the size of the dots, and the larger the point, the greater the correlation coefficient. **(C)** Analysis of the correlation between immune cells signals and the expressions of downregulated Q-ADGs. The correlation coefficient is represented by the size of the dots, and the larger the point, the greater the correlation coefficient.

In the frontal cortex, compared with healthy controls, AD patients had higher B cells naive, Monocytes, Macrophages M1, T cells follicular helper, and Mast cells resting infiltration, while lower B cells memory, T cells CD4 naïve and Dendritic cells activated infiltration (Figure 4). We found that eight types of immune cells were related to 155 Q-ADG in different ways after correlating Q-ADG expressions with immune cells scores measured by CIBERSORT (Supplementary Table 5). T cells CD4 naïve and T cells follicular helper had the most of the number of Q-ADGs, with 89 Q-ADGs and 82 Q-ADGs respectively. Thirty-nine Q-ADGs that showed a correlation with Dendritic cells activated. However, respectively, there are six, one, six, eight, and four Q-ADGs correlated with B cells naïve, Monocytes, Macrophages M1, B cells memory, and Mast cells resting. Among 155 Q-ADGs, 17 Q-ADGs such as STAT3, SMAD3, RAF1, CD81, RORA, ABL1, and HDAC9, correlated with more than three types of immune cells. These results proposed that quercetin may have an important and direct role in regulating immune infiltration, and was more likely to be responsible for regulation of neutrophil infiltration in the frontal lobe of AD.

### Cortex-related quercetin-associated-Alzheimer’s disease-associated genes

**FIGURE 5:**
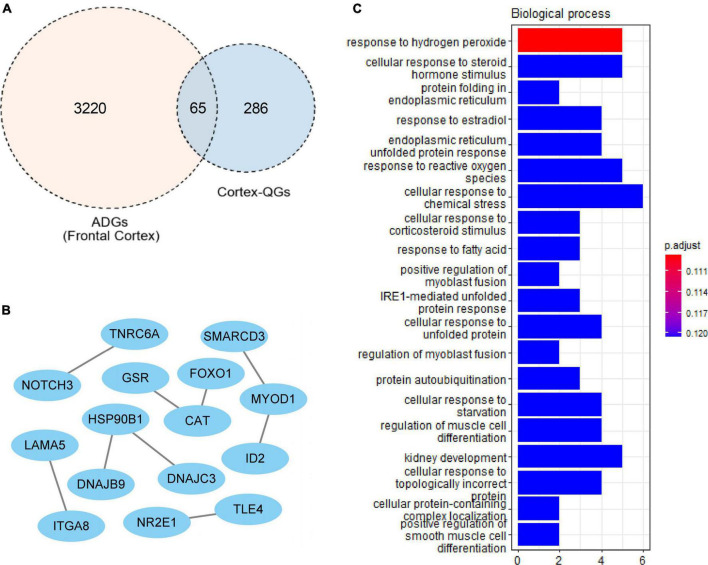
Cortex-related quercetin-associated Alzheimer’s disease-associated genes (Q-ADGs). **(A)** ADGs, cortex-related QGs, and cortex-related Q-ADGs are shown in Venn diagram. **(B)** Cortex-related Q-ADGs interact each other are shown in the PPI network. **(C)** GO enrichment analysis shows the biological process of cortex-related Q-ADGs.

A total of 227 cortex-related QGs were identified (Supplementary Table 6), such as DYRK1α, nqo1 and NGF. We intersected ADGs with cortex-related QGs, and at last found 65 cortex-related Q-ADGs, which was then utilized to conduct the PPI analysis. The PPI network demonstrated that NOTCH3, FOXO1, DNAJC3, CAT, GSR, and ID2 played a critical role in the network, indicating that these core genes might be important targets of quercetin that acted directly in the AD patients’ frontal cortex. According to the GO analysis, response to hydrogen peroxide, protein folding in endoplasmic reticulum, response to reactive oxygen species and other biological processes were enriched within the cortex-related Q-ADGs (Figure 5 and Supplementary Table 6).

### Undetermined quercetin-associated-Alzheimer’s disease-associated genes

**FIGURE 6:**
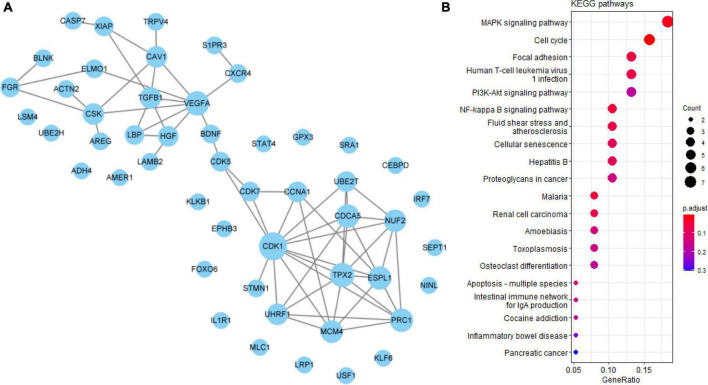
Undetermined quercetin-associated Alzheimer’s disease-associated genes (Q-ADGs). **(A)** PPI network of undetermined Q-ADGs. **(B)** The pathways of undetermined Q-ADGs are shown in KEGG enrichment analysis.

VEGFA, CDK1, and TGFB1 were identified as core genes in the PPI network of 50 undetermined Q-ADGs, indicating that it is possible that quercetin regulates these functionally related genes as well. In addition to the undetermined Q-ADGs, KEGG pathways analysis demonstrated that these genes were enriched in the pathways such as NF-kappa B signaling pathway, MAPK signaling pathway, and apoptosis (Figure 6).

### Severity-related quercetin-associated-Alzheimer’s disease-associated genes

**FIGURE 7:**
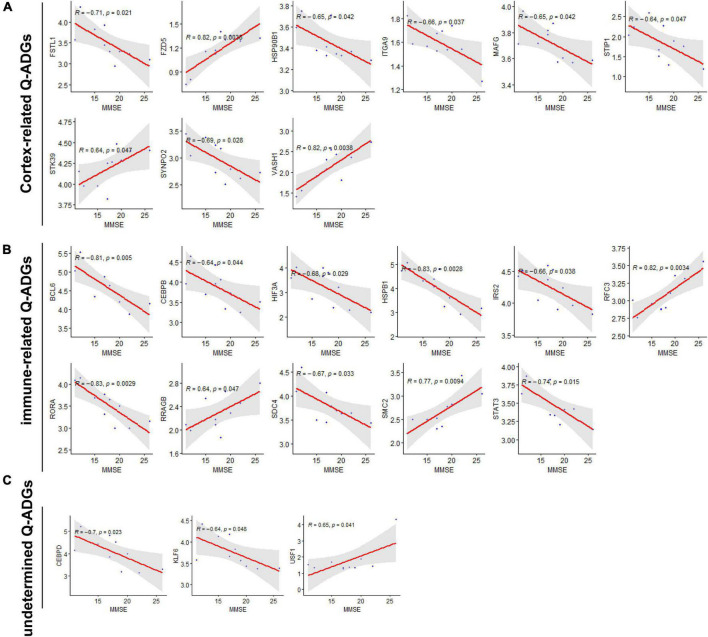
Severity-related quercetin-associated Alzheimer’s disease-associated genes (Q-ADGs). **(A)** Cortex-related Q-ADGs, **(B)** immune-related Q-ADGs, and **(C)** undetermined Q-ADGs. Analysis of the correlation between MMSE scores (11–26) and Q-ADG expressions. On the x-axis, MMSE scores are displayed. On the y-axis, gene expression levels are displayed. Every sample is shown as a blue dot representing the level of expression of key genes.

To collect the correlation between Q-ADGs and the MMSE scores (11–26) of 10 AD patients, severity-related Q-ADGs were identified from 23 Q-ADGs with strong correlations to MMSE scores. Among the severity-related Q-ADGs, such as STIP1, FSTL1, HSP90B1, ITGA9, MAFG, and SYNPO2 had a positive correlation with the clinical severity of AD (negative correlation with the MMSE scores), while STK39, VASH1, and FZD5 had negative correlation with the severity. Among the immune-related Q-ADGs, STAT3, IRS2, HSPB1, RORA, CEBPB, BCL6, SDC4, and HIF3A had a positive correlation with the clinical severity of AD, while RFC3, RRAGB, and SMC2 had negative correlation with the severity. Among the undetermined Q-ADGs, CEBPD and KLF6 had a positive correlation with the severity of AD, while USF1 had negative correlation with the clinical severity (Figure 7 and Supplementary Table 7).

### Severity-related quercetin-associated-Alzheimer’s disease-associated genes-miRNAs

**FIGURE 8:**
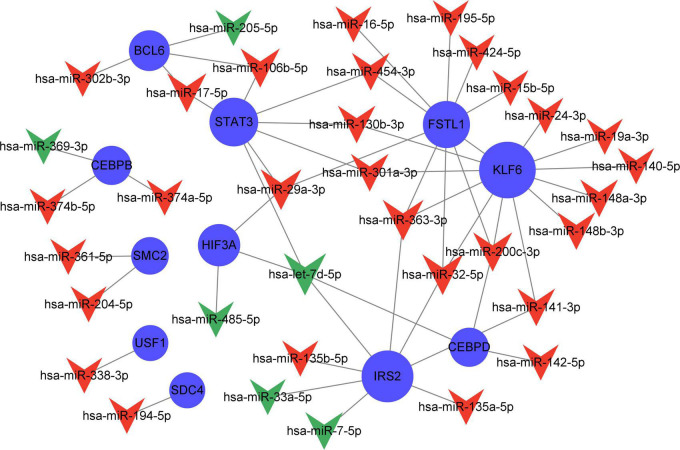
Network of severity-related quercetin-associated Alzheimer’s disease-associated gene (Q-ADG)-miRNAs. The red shapes of V represent upregulated miRNAs in late stage of AD; the green shapes of V represent downregulated miRNAs in late stage of AD; and the blue circles represent the upregulated mRNA with the developing of AD.

Compared with the early stage of AD, in the frontal cortex, 203 miRNAs (169 upregulated and 94 downregulated) were identified to be differently expressed in the late stage. These miRNAs had a correlation with AD severity. There were 38 AD severity-related miRNAs (miR-223-3p, miR-17-5p, miR-26b-5p, etc.) have been identified which target 18 genes (STAT3, IRS2, KLF6, etc.) out of the 23 severity-related Q-ADGs in the group with MMSE scores ranging from 11 to 26 (Figure 8 and Supplementary Table 8).

### Quercetin regulates gene expressions in Alzheimer’s disease cell model

**FIGURE 9:**
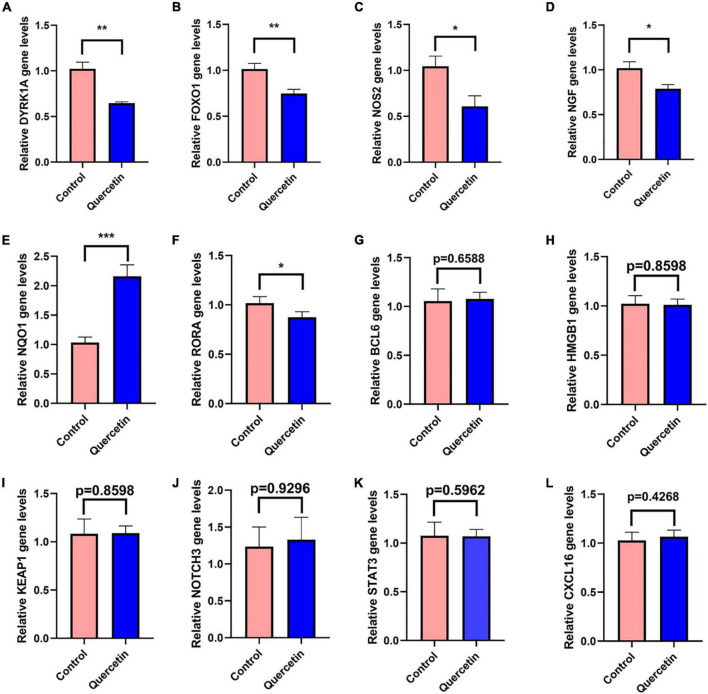
Quercetin regulates gene expressions in the PC12 cell model of AD. QRT-PCR detects the mRNA expressions of DYRK1A **(A)**, FOXO1 **(B)**, NOS2 **(C)**, NGF **(D)**, NQO1 **(E)**, NOTCH3 **(F)**, BCL6 **(G)**, HMGB1 **(H)**, KEAP1 **(I)**, RORA1 **(J)**, STAT3 **(K)**, and CXCL16 **(L)** genes in the Control (Aβ_25–35_ + saline solution) and Quercetin (Aβ_25–35_ + quercetin) groups. *P < 0.05; **P < 0.01; ***P < 0.001.

In the PC12 cell model of AD, 12 selected genes more frequently referred to AD by previous studies among all the genes identified by network pharmacology, DYRK1A, FOXO1, NOS2, NGF, NQO1, NOTCH3, BCL6, HMGB1, KEAP1, RORA1, STAT3, and CXCL16, were detected by the qRT-PCR with or without 80 μM quercetin. The primer sequences for qRT-PCR were shown in Supplementary Table 9. Quercetin significantly downregulated the mRNA levels of DYRK1A, FOXO1, NOS2, NGF, and RORA while quercetin significantly upregulated NQO1 mRNA level (Figure 9). The expression levels of the remaining 6 genes were not affected by quercetin.

## Discussion

**FIGURE 10:**
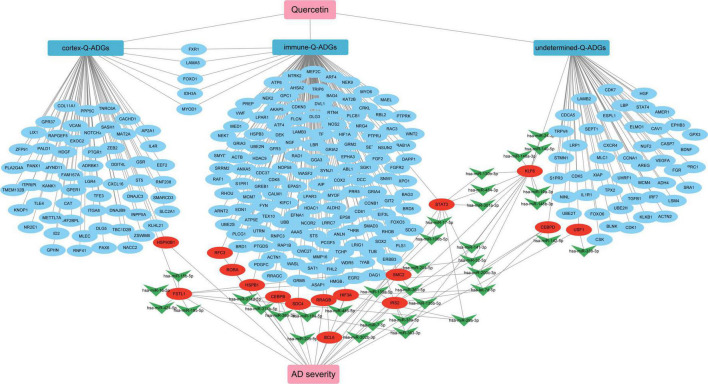
The pharmacological mechanism of quercetin in Alzheimer’s disease (AD) patients is depicted in a schematic diagram. In AD patients, severity-related quercetin-associated AD-associated genes (Q-ADGs) are shown in the red ellipses; the blue ellipses show Q-ADGs which are not associated to MMSE scores; and miRNAs are shown in the green shapes of V.

In the elderly, as the most prevalent type of dementia, AD presents a number of challenges to those who suffer from it (Babatope et al., 2021). Quercetin was recently found to be an anti-inflammatory, antioxidant, analgesic, and perhaps therapeutic agent for COVID-19 in the prevention and neuroprotection of histopathology in AD animal models, but its efficacy in human brain tissue has not been established (Saeedi-Boroujeni and Mahmoudian-Sani, 2021). The purpose of this research was to develop network pharmacology to discover potential quercetin medication targets in the CNS of AD patients. We discovered that quercetin may ameliorate pathological events by altering astrocytes, microglia, the infiltration of B cells naive, T cells follicular helper, Monocytes, Macrophages M1, Mast cells resting, B cells memory, T cells CD4 naïve and Dendritic cells activated in the frontal cortices, biological process of axonogenesis, glial cell differentiation, gliogenesis, axon guidance, neuron projection guidance, KEGG pathway of MAPK signaling pathway, axon guidance, PI3K-Akt signaling pathway, neurotrophin signaling pathway, and miRNA interactions, among others (Figure 10).

DYRK1A gene was involved in neuronal development and serves a number of functions in the adult CNS (Park et al., 2012; Smith et al., 2012; Pathak et al., 2018). DYRK1A mRNA and protein level were significantly upregulated in AD patients brains, including in the hippocampus (Kimura et al., 2007; Velazquez et al., 2019). DYRK1A overexpression may contribute to the synaptic dysfunction and cognitive decline in AD and Down syndrome patients (Park et al., 2012; Smith et al., 2012; Pathak et al., 2018). By phosphorylating PS1 and reducing phosphatidylcholine concentrations, upregulated DYRK1A may contribute to AD pathogenesis (Ryu et al., 2010; Hijazi et al., 2017). Up-regulated DYRK1A downregulate neprilysin which was a major Aβ-degrading enzyme to reduce the pathological process of Aβ aggregation in AD (Kawakubo et al., 2017). DYRK1A increased phosphorylation of tau, APP, and PSEN1, which are three key proteins in AD pathologic process (Wegiel et al., 2011; Branca et al., 2017). In several experimental models of systemic autoimmunity and mucosal inflammation, T regulatory cells (Treg) induced by DYRK1A inhibitors significantly reduced inflammation (Khor et al., 2015). Normalizing DYRK1A gene expression in a mouse model improves numerous AD phenotypes (García-Cerro et al., 2017). The qRT-PCR results in the present study have revealed that quercetin significantly downregulated DYRK1A mRNA expression in AD cell model (*p* < 0.005) (Figure 9A). Inhibition of DYRK1A hyperactivity in AD by quercetin in the brain may pave the way for therapies on cognitive decline in AD patients (Smith et al., 2012; Branca et al., 2017; Pathak et al., 2018).

Oxidative stress and AD are linked together by forkhead box O1 (FOXO1) (Paroni et al., 2014; Zhang W. et al., 2020). FOXO1 overexpression reduced both tau phosphorylation and Aβ expression (Zhang W. et al., 2020). FOXO1 has been shown to activate autophagy by suppressing mTOR (Zhao et al., 2010). A signaling pathway involving IL-7/CD127 was activated by FOXO1, which enhanced Treg cell proliferation (Cai et al., 2017). FOXO1 overexpression reduces inflammation and boosts anti-oxidative capability (Huang et al., 2019). However, contrary to the result in decidualized endometrial stromal cells (Kusama et al., 2021), we found that quercetin significantly decreases FOXO1 mRNA level in AD cell model (*p* < 0.005) (Figure 9B), which indicates that FOXO1, downregulated by quercetin, may play a bad role.

In AD, the inducible form of nitric oxide synthase (NOS2) was increased (Kummer et al., 2012). There has been a connection between NOS2 and the risk of AD or dementia associated with Lewy bodies (Singleton et al., 2001). The activation of the inducible NOS2, which results in increased NO production, which partly leads to the inflammatory response in AD (Kummer et al., 2011). NOS2 isoforms associated with inflammation in brain glial cells are believed to contribute to neurological disorder etiology and progression (Murphy et al., 2002). There was a significant TH17 immune response increase, along with matrix metalloproteinase-9 expression, associated with increased expression of inducible NOS2 (Sodenkamp et al., 2011). NOS2-specific inhibitors diminish inflammation in mice (Weinberg, 2000). The qRT-PCR results in the present study have revealed that quercetin significantly downregulated NOS2 mRNA expression in AD cell model (*p* < 0.05) (Figure 9C). There was a significant reduction in the amount of 3’-nitrotyrosine Aβ (3NTyr^10^-Aβ), overall Aβ deposition and cognition impairments in APP/PS1 animals with a NOS2 deficiency or with oral therapy with the NOS2 inhibitor (Kummer et al., 2011; Irwin et al., 2016).

NQO1, having antioxidant and anti-inflammatory function (SantaCruz et al., 2004; Mokarizadeh et al., 2020), is declined in AD patients’ and mice’s brains (Torres-Lista et al., 2014; Osama et al., 2020; Wang et al., 2020). The NQO1 produces anti-oxidative types of ubiquinone and vitamin E, which contribute to antioxidant protection as well as dopaminergic neuronal tolerance to chronic oxidative damage (Ross et al., 2000; Xu et al., 2019). NQO1 is modulated by Nrf2 gene which contributes to the maintenance of cellular redox homeostasis, regulates inflammation and provides neuroprotection against both Aβ and p-tau (Osama et al., 2020). The qRT-PCR results in the present study have revealed that quercetin significantly upregulated NQO1 mRNA expression in AD cell model (*p* < 0.001) (Figure 9E). Increased NQO1 activity may be neuroprotective for AD patients (Osama et al., 2020).

In PC12 cells, Nerve growth factor (NGF) increases APP mRNA levels through a process that most likely includes Ras activation, and is likely regulated by particular APP promoter sequences (Villa et al., 2001). In AD patients, NGF level is markedly higher in cerebrospinal fluid (CSF), and cholinergic neuron target areas such as the hippocampus and cortex (Hock et al., 2000; Fahnestock et al., 2001; Du et al., 2018). The qRT-PCR results in the present study have revealed that quercetin significantly downregulated NGF mRNA expression in AD cell model (*p* < 0.05) (Figure 9D). Although some studies suggested that NGF prevented cholinergic degeneration and memory deficits (Wu et al., 2004; Fjord-Larsen et al., 2010), proper reduction of the hyperactive NGF by quercetin in AD might benefit for AD patients.

Three immune cells demonstrated significant differences *via* immune infiltration analysis: T cells CD4 naïve, T cells follicular helper, and Dendritic cells activated. Respectively, 101 gene targets were related to immune cells among immune-related Q-ADGs. Up-regulated genes (STAT3, etc.) were positively correlated with T cells CD4 naïve, and negatively correlated with T cells follicular helper and Dendritic cells activated. On the opposite, down-regulated genes (SMAD3, etc.) were negatively correlated with T cells CD4 naïve, and positively correlated with T cells follicular helper and Dendritic cells activated. We proposed that these up- or down-regulated genes had a more direct association with immunological infiltration. COX-2 is increased and has been confirmed to participate in the pathogenesis of AD. These up- or down-regulated genes were consistent with the increase of the astrocyte-associated genes in the brain of AD patients in comparison with controls. An immune cell-regulated enzyme in the brain, cyclooxygenase-2 (COX-2), is produced in neuron as a result of synaptic excitatory activity and inflammation (Woodling et al., 2016). Furthermore, COX-2 can be downregulated by quercetin (Srivastava and Srivastava, 2019). Therefore, by targeting COX-2, quercetin might regulate immune infiltration in the CNS. It is required for the innate immune response against inflammation to be initiated by ROR-alpha (RORA), a transcription factor of nuclear receptors (García et al., 2015). RORA expression is distinctly upregulated in the AD brain (Acquaah-Mensah et al., 2015), consistent with our study the trend of RORA being present in the brain at higher levels in late stage of AD than in early stage of AD. Furthermore, we found RORA was negatively associated with AD clinical severity (MMSE 11-26) (Figure 7B). Overexpression of RORA ameliorated inflammatory damage in the mouse models (Oh et al., 2019). However, the qRT-PCR results in the present study have revealed that quercetin significantly downregulated RORA mRNA expression in AD cell model (*p* < 0.05) (Figure 9J), which may have a negative impact on AD pathological process *via* the enhancement of immune response.

In many neurodegenerative diseases, such as AD and Parkinson’s disease, glia dysfunction contributes to CNS pathology. Neuroinflammation generated by hyperactive glia cells is a significant feature of AD and a possible target for therapy (An et al., 2020). Gene Ontology analysis indicated glial cell differentiation is a significant biological process, where quercetin may exert pharmacological effects.

In the present study, Severity-related Q-ADGs and Severity-related Q-ADG-miRNAs were divided into the part of the normal to moderate dementia group (MMSE 11–26), or severe dementia group (MMSE 0–10) was excluded, because we believe patients with severe dementia may be disturbed by other complex basic diseases, such as cerebrovascular disease, infections, cardiovascular disease, and metabolic diseases, which might exert a higher influence on gene expressions than the pathologies of AD. Otherwise, rather than the whole course of AD, the association between Q-ADG expression levels and AD severity may arise only during certain illness episodes. For example, in the early stages of AD, some genes may correlate favorably with clinical severity, but they might not correlate, or they might be negatively correlated, in the intermediate or late stages. As a result, a stratified analysis of AD patients at various phases of the illness should be conducted in the grounds of a thorough assessment of MMSE scores.

## Data availability statement

The datasets presented in this study can be found in online repositories. The names of the repository/repositories and accession number(s) can be found in the article/Supplementary material.

## Author contributions

CW conducted the data collection. CW and RX designed the study. CW, SL, and YZ conducted the processing and analyzing of the image data. WC and CL conducted the statistical analyses. All authors were involved in the interpretation of the data, writing the manuscript, reviewing the final version of this study, and approved the submitted version.

## Footnotes

1
https://www.cellbank.org.cn/search-detail.php?id=256

## Abbreviations

AD: Alzheimer’s disease
DYRK1A: dual specificity tyrosine phosphorylation regulated kinase-1A
A β: amyloid β -peptide
3NTyr10-A β: 3 ′ -nitrotyrosine A β
APP: amyloid precursor protein
EAE: experimental autoimmune encephalomyelitis
FAD: familial Alzheimer’s disease
DEG: differentially expressed gene
CNS: central nervous system
QGs: quercetin-associated genes
ADGs: AD-associated genes
Q-ADGs: quercetin-associated ADGs
PPI: protein-protein interaction
MMSE: mini-mental state examination
FOXO1: forkhead box O1
BBB: blood–brain barrier
HPLC: high performance liquid chromatography
DMEM: Dulbecco’s modified eagle medium
GEO: gene expression omnibus
Treg: T regulatory
qRT-PCR: quantitative real-time polymerase chain reaction
mTOR: mechanistic target of rapamycin
PS1: presenilin 1
NOS2: nitric oxide synthase
NQO1: nicotine-adenine diphosphonucleotide, quinone oxidoreductase 1.
