# Supplementary figures and images for: Network pharmacology identify intersection genes of quercetin and Alzheimer’s disease as potential therapeutic targets
Source: Front Aging Neurosci. 2022 Aug 23;14:902092. doi: 10.3389/fnagi.2022.902092 (PMC9447902; doi:10.3389/fnagi.2022.902092)

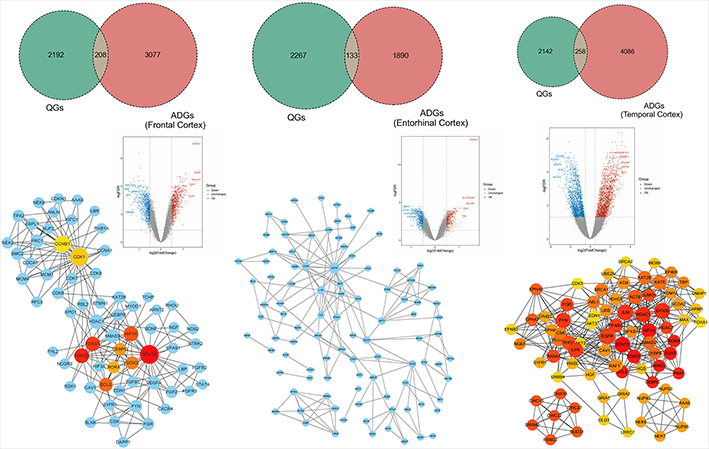

Supplement: Supplementary file 1 [file Image_1.TIF]
